# Supplementary material for: Pilot Testing of a Patient Decision Aid for Adolescents with Severe Obesity in US Pediatric Weight Management Programs within the COMPASS Network
Source: Int J Environ Res Public Health. 2019 May 20;16(10):1776. doi: 10.3390/ijerph16101776 (PMC6572315; doi:10.3390/ijerph16101776)
Supplement: Supplementary file 1 [file ijerph-16-01776-s001.pdf]

Supplementary Materials

Table S1. Medical provider feedback questionnaire.
Following data collection, responses to these 7 questions were elicited from the medical provider who primarily used the tool at each site (n=6) via email.

1) Did your site make any changes to the original SDM tool prior to use (excluding logistical edits like program name/contact info)?
2) What did you like about the SDM tool itself (e.g. content, structure)?
3) What did you like about using the SDM tool with patients/families?
4) What did you not like about the SDM tool itself (e.g. content, structure)?
5) Were there any barriers to using the SDM tool with patients/families in clinic?
6) What changes would you suggest to improve the SDM tool?
7) Do you plan to use the SDM tool in the future as an aid to discuss treatment options for severely obese adolescents?

Figure S1. Patient/family survey.

This 13-question survey was completed by patients/families (n=31) immediately after the shared decision making tool was presented by the medical provider either online using SurveyMonkey® or using a paper copy. It is available to view online at: https://www.surveymonkey.com/r/bariatricsdm.

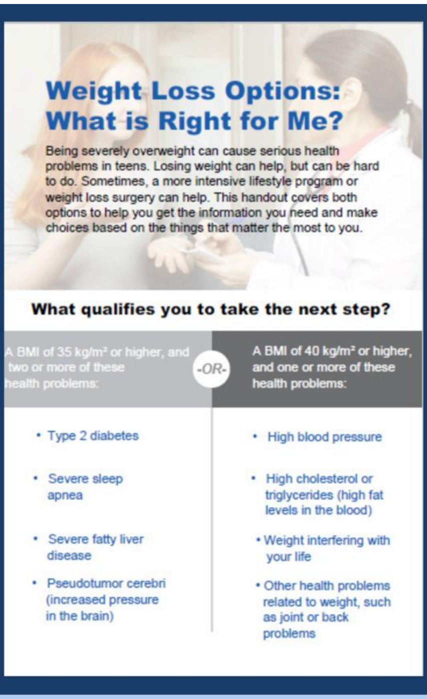

Shared Decision Making-Bariatric Study

Shared Decision Making Survey for Bariatric Surgery or Lifestyle Management Alone

Welcome to our survey to evaluate our Shared Decision Making Tool. This educational tool is designed to help our patients and their families to decide between two types of treatment, surgical weight loss (bariatric surgery) with intensive lifestyle changes and intensive lifestyle changes alone. We are asking for the patient and his/her family to respond to this survey about the patient's visit and health, so please work together. All the questions are about today's visit with your medical provider. Thank you so much for completing this survey.

* **1. Parental Permission: We are conducting a research study to understand whether a Shared Decision Making Tool (educational handout) can be helpful in helping families decide between Bariatric Surgery and Intensive lifestyle management alone for adolescents who have participated in a Pediatric Weight Management Program. Your child's participation in this study is voluntary. If you do not want your child to take this survey, his/her medical care will not be affected. If your child chooses to take the survey, his/her answers will be completely private and confidential. The answers to the survey will never be connected back to your child.**

If you agree to allow your child to participate in this study, please click "yes" to the question below.

◯ Yes

◯ No

* **2. Child's Assent: We are conducting a research study to understand whether a Shared Decision Making Tool (educational handout) can help families decide between Bariatric Surgery and Intensive lifestyle management alone. Your participation in this study is voluntary. If you do not want to take this survey, your medical care will not be affected. If you choose to take the survey, your answers will be completely private and confidential and never be connected back to you.**

If you agree to participate in this study, please click "yes" to the question below.

◯ Yes

◯ No

3. Did your clinician show you the Shared Decision Making Tool (educational handout pictured above) during your visit?

◯ yes

◯ No

* **4. Which pediatric weight management program does your child (you) attend?**

5. How much effort was made to help you understand your (child's) health issues? (choose one answer)

No effort at all 0	1	2	3	4	5	6	7	8	Every effort was made 9
○	○	○	○	○	○	○	○	○	○

6. How much effort was made to listen to the things that matter most to you about your (child's) health issues? (choose one answer)

No effort at all 0	1	2	3	4	5	6	7	8	Every effort was made 9
○	○	○	○	○	○	○	○	○	○

7. How much effort was made to include what matters most to you in choosing what to do next? (choose one answer)

No effort at all 0	1	2	3	4	5	6	7	8	Every effort was made 9
○	○	○	○	○	○	○	○	○	○

8. Did you discuss Intensive Lifestyle Changes to treat your (child's) weight ?

○ Yes

○ No

9. Did you discuss Bariatric Surgery (Surgical Weight Loss) to treat your (child's) weight ?

○ Yes

○ No

10. Do you know the benefits and risks of each option?

- ◯ Yes
- ◯ No

11. Are you clear about which benefits and risks matter most to you and your child?

- ◯ Yes
- ◯ No

12. Do you have enough support and advice to make a choice?

- ◯ Yes
- ◯ No

13. Do you feel sure about the best choice for you (your child)?

- ◯ Yes
- ◯ No

Thank You for Completing the Survey

Prev Done

Powered by
SurveyMonkey
See how easy it is to create a survey.
